# Human CD8^+^CD28^−^ T suppressor cells expanded by common gamma chain (γc) cytokines retain steady allospecific suppressive capacity in vivo

**DOI:** 10.1186/s12865-020-00354-z

**Published:** 2020-04-29

**Authors:** Guihuan Liu, Yuming Yu, Fu Feng, Ping Zhu, Hua Zhang, Danni Zhang, Xiaoqiang Feng, Zedan Zhang, Yanjun Liu

**Affiliations:** 1 Department of Immunology, School of Basic Medical Science, Southern Medical University, No.1023-1063 Shatainan Road, Guangzhou, 510515 Guangdong China; 2 Department of Urology, Guangdong Provincial People’s Hospital (Guangdong Academy of Medical Sciences), No.106 Zhongshan 2nd Road, Guangzhou, 510080 Guangdong China

**Keywords:** CD8^+^CD28^−^ T suppressor cells, Common gamma chain cytokines, Transplant tolerance, Alloantigen specific tolerance

## Abstract

**Background:**

CD8^+^CD28^−^ T suppressor (Ts) cells play critical role in transplant tolerance. Our previous study has generated CD8^+^CD28^−^ Ts cells in vitro which exert robust allospecific suppressive capacity in vitro.

**Results:**

CD8^+^CD28^−^ Ts cells were expanded by stimulating human CD8^+^ T cells with allogeneic antigen presenting cells in the presence of the common gamma chain cytokines IL-2, IL-7 and IL-15 in vitro, and were further verified in vitro through day 7 to 11 for their persistency of the allospecific suppressive capacity. When CD8^+^CD28^−^ Ts cells were adoptively transferred into NOG mice, their capacity to inhibit CD4^+^ T cell proliferation in allospecific manner remained potent on 11 days after their injection. The mechanisms for expansion of CD8^+^CD28^−^ Ts cells by the common gamma chain cytokines were investigated. These included promoting CD8^+^CD28^−^ T cells proliferation, converting CD8^+^CD28^+^ T cells to CD8^+^CD28^−^ T cells and decreasing CD8^+^CD28^−^ T cell death. Furthermore, the expanded CD8^+^CD28^−^ Ts cells showed upregulation of the co-inhibitory molecule Tim-3 and down-regulation of the cytotoxic molecule granzyme B.

**Conclusions:**

In summary, these results demonstrated that the in vitro-expanded human CD8^+^CD28^−^ T cells retained potent allospecific suppressive capacity in vivo and depicted multiple mechanisms for the expansion of Ts cells, which might promote further bench to clinic research.

## Background

Long-term use of non-specific immunosuppressive drugs after transplantation results in generalized immunosuppression in recipients, which may place recipients at the risk of serious complications, such as infection and malignant conditions [1, 2]. Therefore, it is urgent to find novel measures to induce alloantigen specific tolerance after transplantation.

CD4^+^CD25^+^Foxp3^+^ regulatory T cells (Tregs) have been demonstrated to maintain immune tolerance in transplantation and other physiological circumstances [3–5]. Recently, research on another subset of regulatory T cells, CD8^+^CD28^−^ Ts cells, have quickly gained attention in this field. CD8^+^CD28^−^ Ts cells have been documented to promote immune tolerance by suppressing effector T cells responses to alloantigen in transplantation [6–8]. They have also been implicated in chronic viral infections [9], autoimmune diseases [10, 11], cancer [12]. However, relatively simple and rapid methods for generating a sufficient number of CD8^+^CD28^−^ Ts cells are lacking, constituting a main hurdle for further research. In our previous studies, [13] we have expanded large numbers of human CD8^+^CD28^−^ Ts cells in a relatively short period of time by stimulating CD8^+^ T cells with antigen presenting cells (APCs) when supplemented with triple common gamma chain cytokines IL-2, IL-7 and IL-15 in vitro. However, detailed characteristics of the expanded CD8^+^CD28^−^ Ts cells were unclear. The principal function of these population when transferred in vivo was also yet to be examined, as well as the mechanism of the common gamma chain cytokines in the expansion of CD8^+^CD28^−^ T cells awaited more research.

In this study, we show that the in vitro-expanded CD8^+^CD28^−^ Ts cells maintain allospecific suppressive capacity both in vitro and in vivo, and that multiple mechanisms contributed to the in vitro expansion. These findings will facilitate CD8^+^CD28^−^ Ts cell-based therapeutic strategies for clinical transplantation in the future.

## Results

### In vitro-expanded CD8^+^CD28^−^ T cells possess durable suppressive activity at different time points in vitro

We previously described that in vitro-expanded CD8^+^CD28^−^ Ts cells in the presence of donor APCs plus the combination of common gamma chain cytokines IL-2, IL-7, and IL-15 could suppress the proliferation of CD4^+^ T cells in allospecific manner at one time point i.e. 7 days after culture [13]. We then further investigated whether the suppressive capacity of these cells was durable. As described in Methods, in vitro generation of CD8^+^CD28^−^ T cells were set up as described previously [13]. Briefly, Enriched A-CD8^+^ T cells (2 × 10^6^ cells) (CD8^+^ T cells from individual A) were stimulated with B-APCs (1 × 10^6^ cells) (APCs from individual B) (HLA-A, −B, −DR mismatched with individual A) in 24-well plates in the presence of IL-2(20 U/ml), IL-7 (50 ng/ml) and IL-15 (50 ng/ml). After 9 days’ incubation, in vitro-expanded CD8^+^CD28^−^ T cells were enriched. Then, CFSE-labeled A-CD4^+^ T cells (CD4^+^ T cells from individual A) (5 × 10^4^/well) were cocultured with B-APCs (5 × 10^4^/well) or I-APCs (APCs from individual I, HLA-A, −B and -DR fully mismatched with individual A and B) (5 × 10^4^/well) in the presence or absence of in vitro-expanded CD8^+^CD28^−^ T cells (2. 5 × 10^4^/well) in 96-well round bottom plate for 7 days and 11 days. CFSE diluted assay was used to assess the proliferation of CD4^+^ T cells.

**Fig. 1:**
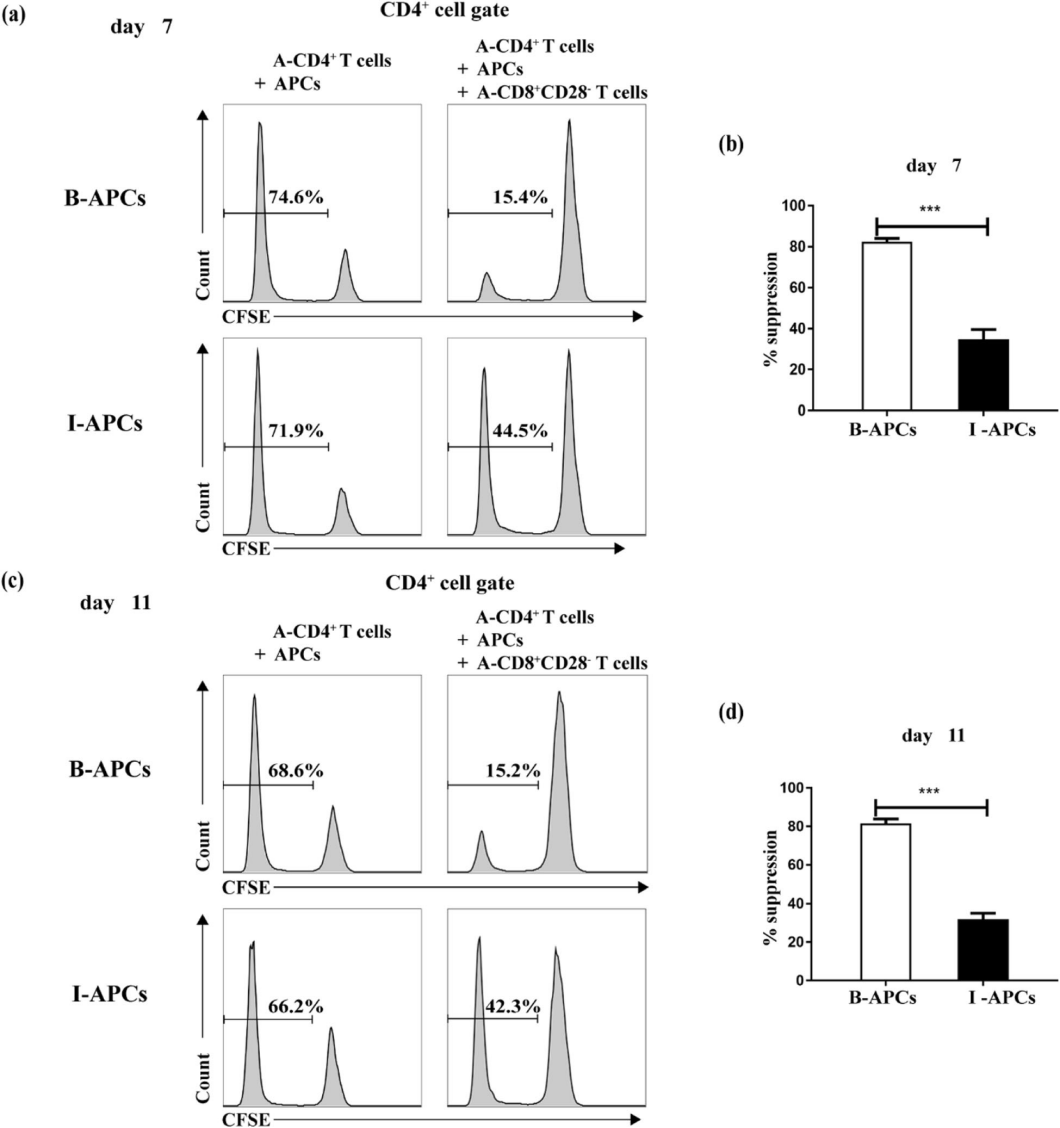
In vitro*-*expanded CD8^+^CD28^−^ T cells possess durable suppressive activity at different time points in vitro*.* The in vitro-expanded CD8^+^CD28^−^ T cells (2.5 × 10^4^/well), which had been primed with B-APCs during the expansion culture, were tested for the ability to suppress proliferation of autologous CFSE-labeled CD4^+^ T cells (5 × 10^4^/well) stimulated with allogenic APCs (5 × 10^4^/well) in vitro for 7 days and 11 days. The top and bottom displayed in the histogram were the data expressed as percentage of CD4^+^ T cell proliferation in the B-APCs (top) or the I-APCs (bottom) stimulating group measured by CFSE dilution from a representative experiment after 7 days (**a**) or 11 days (**c**) of coculture. Statistical analysis displayed in bar graphs showed the suppression percentage of CD4^+^ T cells proliferation by the in vitro-expanded CD8^+^CD28^−^ T cells in the B-APCs or the I-APCs stimulating group after 7 days (**b**) or 11 days (**d**) of coculture (*n* = 3). *** *P*<0.001

As shown in Fig. 1a, following exposure to B-APCs, 74.6% of A-CD4^+^ T cells went on proliferating on the 7th day, but in the presence of CD8^+^CD28^−^ T cells, the proliferating percentage of A-CD4^+^ T cells sharply decreased to 15.4%; in contrast, when exposing to I-APCs, the proliferating percentage of A-CD4^+^ T cells also dropped to a much less extent, from 71.9 to 44.5%. As shown in Fig. 1b, the suppression percentage (% suppression) of in vitro-expanded CD8^+^CD28^−^ T cells were more potent in the B-APCs exposure group compared with the I-APCs group (82.36 ± 2.69% vs.34.80 ± 8.22%, *P* < 0.001). Similarly, on the 11th day, in vitro-expanded CD8^+^CD28^−^ T cells exhibited more vigorous suppressive ability in the B-APCs exposure group compared to the I-APCs group (Fig. 1c). The suppression percentage of CD8^+^CD28^−^ T cells in the two groups showed statistical significance (81.57 ± 4.22% vs.31.87 ± 5.46%, *P* < 0.001) (Fig. 1d).

These results indicated that in vitro-expanded CD8^+^CD28^−^ T cells possessed durable ability to quell CD4^+^ T cells proliferation in an alloantigen specific manner.

### In vitro-expanded CD8^+^CD28^−^ T cells maintain suppressive activity in vivo

**Fig. 2:**
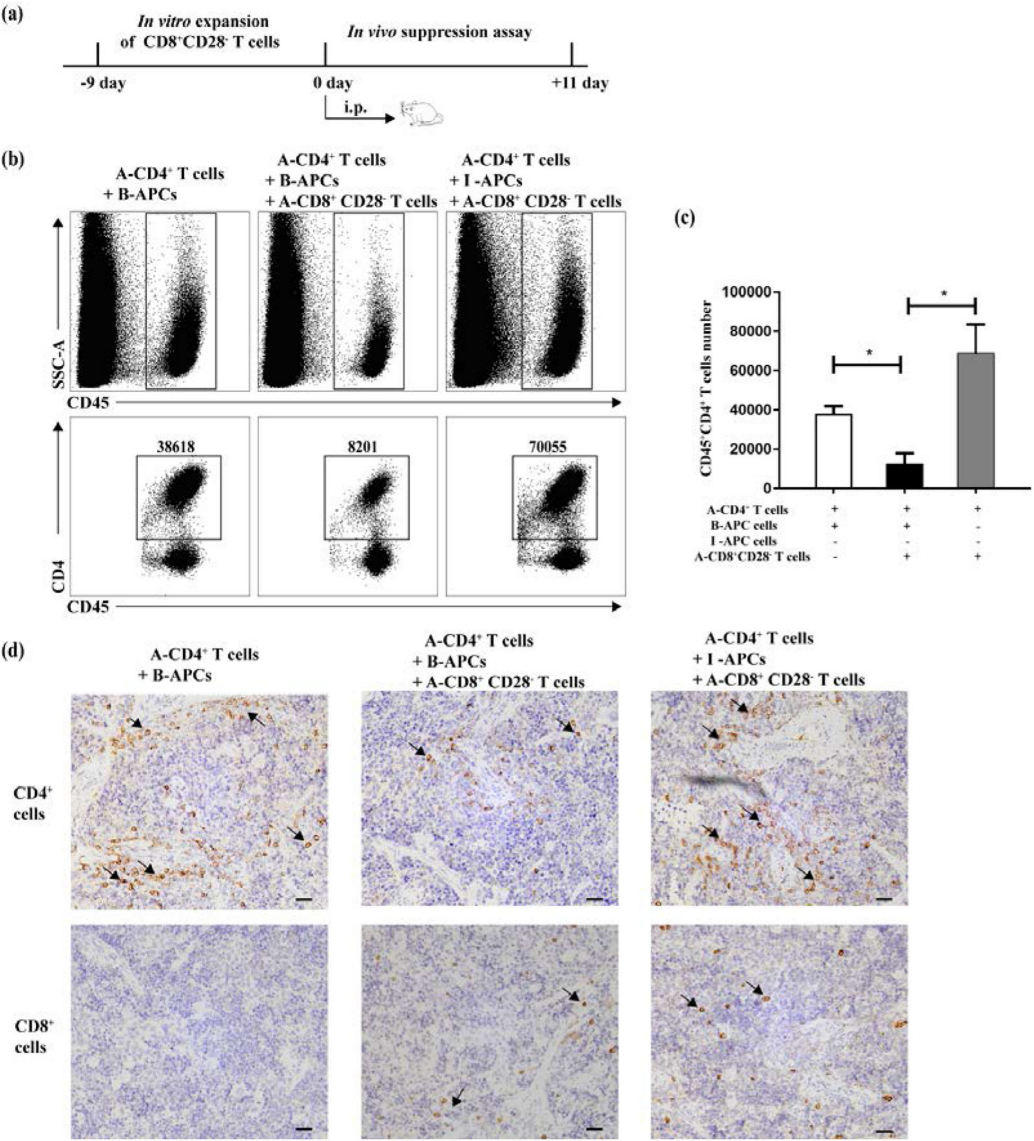
In vitro-expanded CD8^+^CD28^−^ T cells maintain allospecific suppressive activity in vivo*.***a** Experimental procedure: purified CD8^+^ T cells were expanded in vitro in the presence of allogenic APCs plus the combination of the cytokines IL-2, IL-7, and IL-15 for 9 days. The mixture of human cells, which were the same as the in vitro suppression assay (4 × 10^6^ human CD4^+^ T cells were first mixed with an equal number of APCs either from B-APCs or I-APCs, and then combined with 2 × 10^6^ in vitro expanded human CD8^+^CD28^−^T cells in a total volume of 1.5 ml of PBS.), were injected into abdominal cavity of NOG mice. The mice would be sacrificed after 11 days of injection. **b** Representative dot plots demonstrated the existence of human cells by the expression of the molecule CD45, whereas the total cell number of CD45^+^CD4^+^ cells was counted for assessing the inhibitory function exerted by in vitro-expanded CD8^+^CD28^−^ T cells. **c** Aggregated data from three independent experiments using different stimulator-responder pairs were shown (*n* = 3). **d** The immunohistochemical images represented human CD4^+^ and CD8^+^ T cells (As indicated by the arrow) infiltrating in the spleen and were representative of data from all animals. Magnification: × 400. (*n* = 3)

Next, we investigate the stability of the allogeneic suppressive capacity in vivo for the in vitro-expanded CD8^+^CD28^−^ Ts cells. Corresponding to cell components from in vitro experiments, a mixture of human cells was administered into NOG mice by intraperitoneal injection. Eleven days after the injection, mice were sacrificed and human CD4^+^ T cells were investigated in the spleen of mice by flow cytometry and immunohistochemistry to determine if CD8^+^CD28^−^ T cells could confer a local state of immunosuppression (Fig. 2a).

Compared to simply transferring the B-APC group, the absolute number of human CD4^+^ T cells (CD45^+^CD4^+^ cells) as an indicator of inhibition of APCs-induced T cell proliferation by CD8^+^CD28^−^ T cells, was markedly reduced in the group co-transferred with CD8^+^CD28^−^ T cells, whereas the suppression was not seen when I-APCs were co-transferred as stimulator (Fig. 2b, c). The tendency was in agreement with observations from in vitro suppression assay, and similar results were also obtained from additional stimulator-responder pairs. Moreover, the immunohistochemical images demonstrated human cells, both CD4^+^ cells and CD8^+^ (CD8^+^CD28^−^) T cells, were consistently homed to spleen and remained alive at least 11 days after injection (Fig. 2d). In addition, the result visually revealed a smaller number of human CD4^+^ T cell infiltration in the spleen in B-APCs plus CD8^+^CD28^−^ T cells group when compared with B-APCs group and I-APCs plus CD8^+^CD28^−^ T cells group. These differences suggested the suppression by CD8^+^CD28^−^ T cells were antigen specific.

### CD8^+^CD28^−^ T cells are induced by multiple mechanisms triggered by the common gamma chain cytokines

Although we showed CD8^+^CD28^−^ Ts cells could be amplified the in vitro culture system with the common gamma chain cytokines IL-2, IL-7, and IL-15, the mechanism remained obscure.

**Fig. 3:**
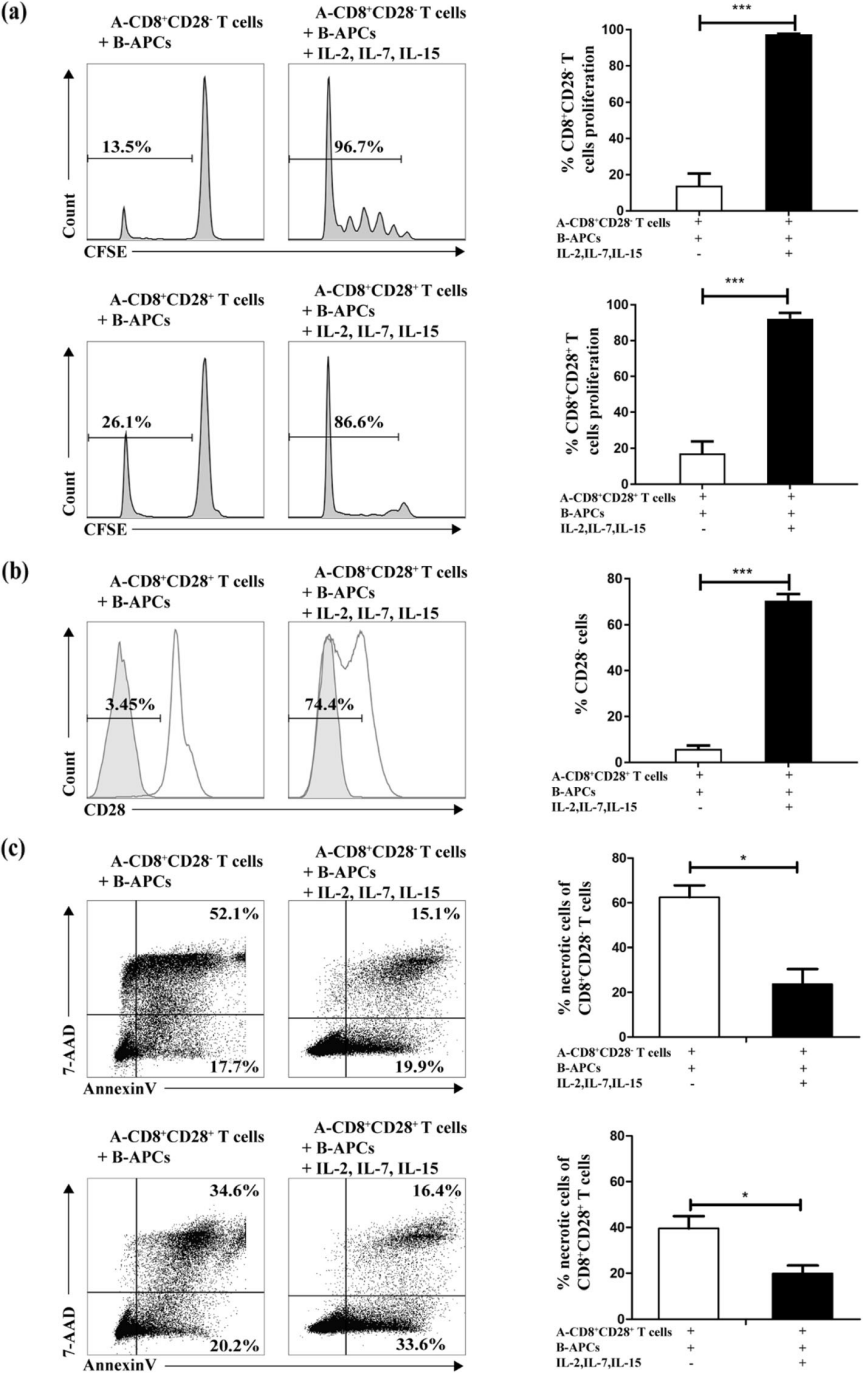
CD8^+^CD28^−^ T cells are induced by multiple mechanisms triggered by the common gamma chain cytokines. Some possible mechanisms accounting for the expansion of CD8^+^CD28^−^ T cells by the common gamma chain cytokines IL-2, IL-7, and IL-15 were elucidated. **a** Proliferation. The proliferation of purified CD8^+^CD28^−^ and CD8^+^CD28^−^ T cells were measured in the presence or absence of the combination of common gamma chain cytokines. **b** Transformation. The percentage of CD8^+^CD28^−^ T cells was shown when purified CD8^+^CD28^+^ T cells were cocultured in the presence or absence of the cytokines for 9 days. Isotype control antibody (grey) was used in the flow cytometry. **c** Survival. After 9 days of culture, the percentage of necrotic cells (Annexin V^+^ 7-AAD^+^) in the CD8^+^CD28^+^ and CD8^+^CD28^−^ T cells was assessed by flow cytometry. Aggregated data from three experiments using different stimulator-responder pairs are shown (*n* = 3). *** *P*<0.001,* *P*<0.05

First of all, we examined whether CD8^+^ T cells with diverse expression of CD28 differed in proliferative capacity in the presence of the common gamma chain cytokines for 9 days. To do so, the proliferative capacity of CD8^+^CD28^+^ T cells or CD8^+^CD28^−^ T cells by APC stimulation in the presence of the cytokines were measured by CFSE dilution. An apparent increase in proliferation in both subsets, with respect to control, was observed in response to the common gamma chain cytokines (CD28^−^: 13.87 ± 11.64% vs. 97.33 ± 0.55%, *P* < 0.001; CD28^+^: 17.08 ± 11.49% vs. 92.23 ± 5.55%, *P* < 0.001) (Fig. 3a).

We next examined whether the common gamma chain cytokines could promote the transformation of CD8^+^CD28^+^ T cells to CD8^+^CD28^−^ T cells. To do so, CD8^+^CD28^+^ T cells were purified and incubated in coculture system with the common gamma chain cytokines. We observed the generation of large amounts of CD8^+^CD28^−^ T cells during the culture (5.83 ± 3.04% vs. 70.37 ± 5.83%, *P* < 0.001) (Fig. 3b), indicating a progressive decrease of CD28 expression concurrent with the stimulated proliferation of the CD8 ^+^ CD28^+^ T cells.

Lastly, we evaluated the effect of the common gamma chain cytokines on cell survival by staining with Annexin V. There was a considerable necrotic rate for CD8^+^CD28^+^ T cells and CD8^+^CD28^−^ T cells without the stimulation of any cytokine during the period of 9 days’ incubation. However, the addition of the common gamma chain cytokines resulted in a decreasing cell death, particularly of CD8^+^CD28^−^ T cells, and consequently the expansion of CD8^+^CD28^−^ T cells (CD28^−^: 62.43 ± 9.21% vs. 23.63 ± 11.80%, *P* < 0.05; CD28^+^: 39.56 ± 9.21% vs. 19.80 ± 6.15%, *P* < 0.05) (Fig. 3c).

### The phenotypic marker of in vitro-expanded CD8^+^CD28^−^ T cells

**Fig. 4:**
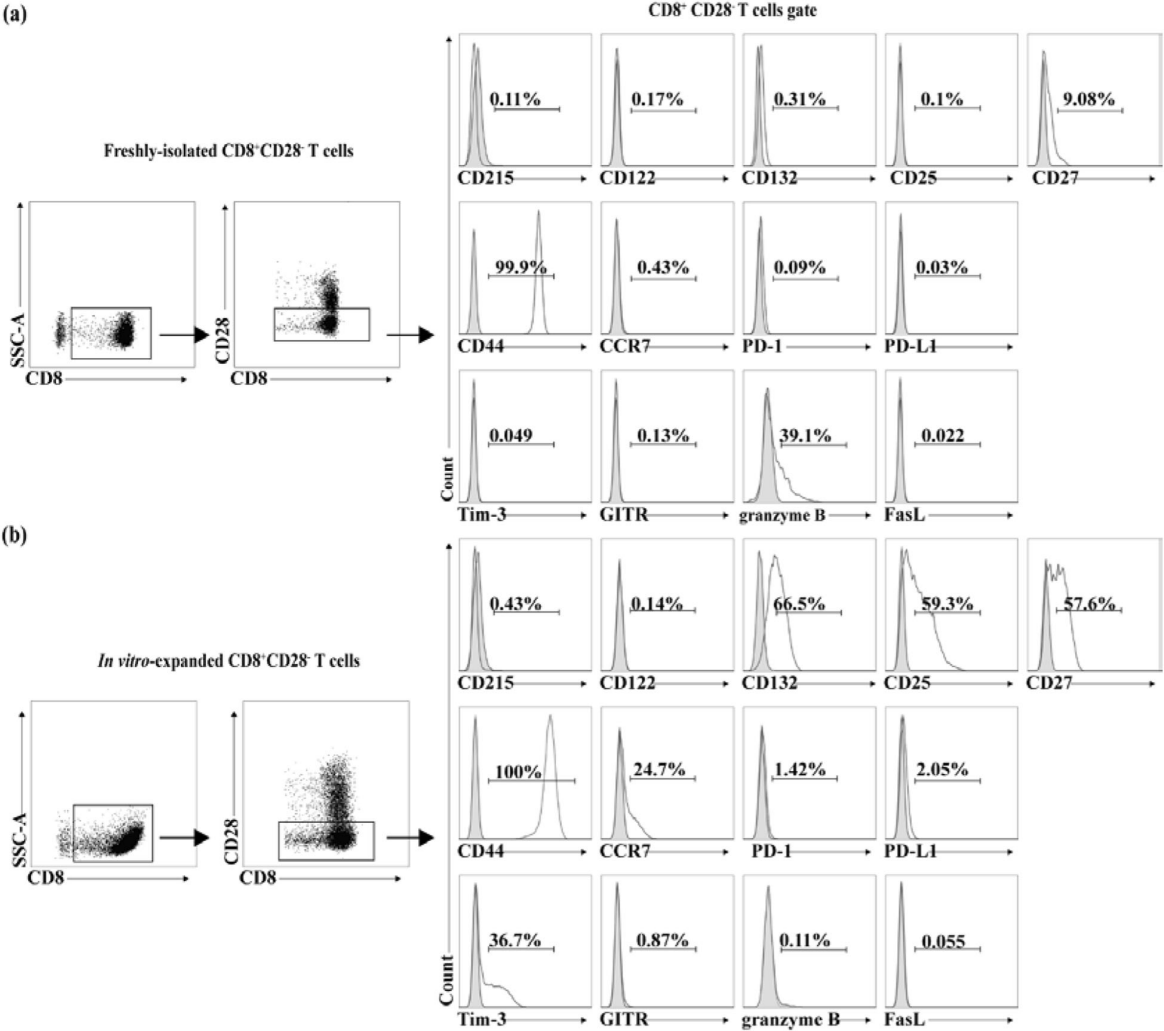
The phenotypic feature of in vitro-expanded CD8^+^CD28^−^ T cells. The in vitro-expanded CD8^+^CD28^−^ T cells were endowed with potent immunomodulatory capacities as suppressor T cells and were distinct in phenotype from CD8^+^CD28^−^ T cells that were freshly purified from human PBMCs. Histogram represented the expression of indicated markers on freshly isolated (**a**) and in vitro-expanded CD8^+^CD28^−^ T cells (**b**), respectively. Control Ab isotype (grey) was included for each staining. Data were representative individual from multiple separate individuals and independent expansions. (*n* = 3)

Cells can sense and respond to cytokines dependent on the expression of cytokine receptors. Using the combination of the cytokines IL-2, IL-7, and IL-15 as stimuli in the culture system, we compared the expression of cytokine receptors on the in vitro-expanded CD8^+^CD28^−^ T cells with those of freshly purified cells, and found that the expression of CD215 (IL-15Rα) and CD122 (IL-2Rβ) remained stable, whereas the expression of the α chain of the IL-2 receptor (CD25) (fresh: 0.19 ± 0.19% vs. expanded: 64.33 ± 4.44%; *P* < 0.001) and the common cytokine receptor γ chain (CD132) (fresh: 1.02 ± 0.77% vs. expanded: 40.16 ± 23.63%; *P* = 0.103) were significantly up-regulated on the expanded CD8^+^CD28^−^ T cells (Fig. 4), indicating stimulation through cytokine receptor CD25 and/or CD132 signal might regulate the expansion of CD8^+^CD28^−^ T cells.

CD8^+^CD28^−^ T cells are heterogeneous populations and can be further divided into distinct subsets according to different combinations of the expression of CD27 [14] and other markers. Recent evidence suggested the loss of CD27 might be closely associated with the terminal differentiation and senescence [14]. Initially, the total percentage of CD8^+^CD28^−^CD27^+^ T cells stood at 9.57 ± 0.64%, but culture with the common gamma chain cytokines after 9 days caused an increase in the cell surface expression (54.90 ± 22.07%, *P* = 0.071) (Fig. 4). Additionally, surface molecules CD44 and CCR7 have been used to broadly distinguish memory cell subpopulations. We found that CD44 expressed highly in both the freshly isolated CD8^+^CD28^−^ T cells and expanded CD8^+^CD28^−^ T cells. Besides, CCR7 did not express in freshly purified CD8^+^CD28^−^ T cells (0.39 ± 0.26%), but the treatment presented in this study led to an increase for CCR7 expression (16.12 ± 7.79%, *P* = 0.073) on the in vitro-expanded CD8^+^CD28^−^ T cells (Fig. 4).

Although exactly how CD8^+^CD28^−^ T cells quell immune responses has been extensively investigated [15], immunomodulatory molecules expressed by immune cells such as programmed death 1 (PD-1) and T-cell Ig mucin domain containing molecule 3 (Tim-3) have emerged as a promising mechanisms to dampen the proliferation of T cell to effectively maintain tolerance. There seem to be a slight rise for PD-1 (fresh: 0.90 ± 0.82% vs. expanded: 1.53 ± 0.86%; *P* = 0.412) and PD-L1 (fresh: 0.18 ± 0.16% vs. expanded: 1.15 ± 0.79%; *P* = 0.166) expression on CD8^+^CD28^−^ T cells during culture. However, a significant number of CD8^+^CD28^−^ T cells after the co-culture displayed increased level of Tim-3 expression (fresh: 0.06 ± 0.02% vs. expanded: 39.90 ± 3.60%; *P* < 0.001). Glucocorticoid–induced TNF receptor (GITR) expressed by regulatory and activated T cells, provides positive co-stimulation for T-cell activation. In our study, no significant difference in expression of GITR (fresh: 0.35 ± 0.41% vs. expanded: 0.82 ± 0.12%; *P* = 0.127) was observed (Fig. 4). On the other hand, cytotoxic function of CD8^+^ T cells might contribute to suppress immune responses. Granzyme B was obviously expressed on freshly isolated CD8^+^CD28^−^ T cells (32.36 ± 20.88%), and its expression was decreased after in vitro-expansion (4.63 ± 3.83%; *P* = 0.144) (Fig. 4). Both CD8^+^CD28^−^ T cells expressed very little Fas ligand (fresh: 0.05 ± 0.07% vs. expanded: 0.07 ± 0.05%; *P* = 0.755) (Fig. 4).

## Discussion

Antigen specific tolerance induction is a valuable treatment for organ transplantation and auto-immune disease. In addition to the inducement of effector T cell anergy and apoptosis [16], immunoregulatory cells, such as CD8^+^ regulatory T cells, have gained increasing attentions. CD8^+^CD28^−^ Ts cells, among CD8^+^ regulatory T cells, are of great potential clinical implications, but the limited number of CD8^+^CD28^−^ Ts cells have been the main hurdle in their clinical application. Our previous study has reported a novel method to induce the rapid expansion of CD8^+^CD28^−^ T cells from naïve CD8^+^ T cells, which were stimulated with donor APCs plus the common gamma chain cytokines IL-2, IL-7 and IL-15 [13]. These expanded CD8^+^CD28^−^ Ts cells efficiently blocked the proliferation of responder CD4^+^ T cells in response to APCs in antigen-specific manner in vitro (Fig. 1a), but the stability of these cells in vivo was not yet clear. Besides, mechanisms of the cytokine-driven expansion of these cells and their phenotypic markers after expansion required further exploration.

In this respect, the proliferation of responder CD4^+^ T cells were inhibited on the 11th day both in vitro and in vivo suppression experiments (Figs. 1c, d and 2), indicating that these CD8^+^CD28^−^ Ts cells were efficient for durable suppression. Although these expanded Ts cells were believed to quell CD4^+^ T-cells responses through direct contact in transwell experiments in vitro [13], some other research suggested that the secretion of certain immunosuppressive cytokines, such as IL-10 [17, 18], might involve the process. Whether the above mechanisms are included in the inhibition experiments in mice in this study still need further research.

As stimulus, the common gamma chain cytokines IL-2, IL-7 and IL-15 multi-dimensionally affected the percentage and the amount of CD8^+^CD28^−^ Ts cells in the expansion cultures *in vitro*. Upon stimulation, the expanded CD8^+^CD28^−^ Ts cells became activated due to the increased levels of CD25 and were more sensitive to IL-2. Moreover, a considerable proportion of γ subunit CD132 was expressed on these Ts cells, but not the β subunit CD122 (Fig. 4), implying that signaling through CD132 might promote CD8^+^CD28^−^ Ts cells expansion and enhance their function. But how CD132-dependent signal generates CD8^+^CD28^−^ Ts cells has yet to be explored.

Up to now, the expansion mechanism for CD8^+^CD28^−^ Ts cells has not been understood clearly. CD8^+^CD28^−^ T cells were generally considered as terminally differentiated, senescent T cells that have undergone many rounds of cell division and show a dramatic decrease in activity or loss of telomerase [19, 20]. Researchers showed that CD8^+^CD28^−^ T cells purified from PBMCs were considered to lack proliferative capability in response to antigen [21, 22]. Interestingly, Chiu WK, et al. noted that CD8^+^CD28^−^ T cells could be endowed with potent proliferative capacities while certain cytokines, such as IL-15, were provided [23]. In this research, both CD28^+^ and CD28^−^ populations of CD8^+^ T lymphocytes were induced to proliferate, especially CD8^+^CD28^−^ Ts cells that underwent extensive cell proliferation in the expanded system (Fig. 3a). Compared to the so called senescent CD8^+^CD28^−^ T cells freshly separated from peripheral blood, CD8^+^CD28^−^ Ts cells expanded in this research have different phenotypes (Fig. 4a and b). Obviously, they belong to different cell subgroups. Further immunophenotypic examination and functional experiment are needed to depict their characteristics. Furthermore, our result confirmed that the combination of cytokines could promote the transformation of CD8^+^CD28^+^ T cells to CD8^+^CD28^−^ T cells (Fig. 3b). The cues determining the switch were not well known, although the existence of IL-2, IL-15 and other key cytokines in the microenvironment may be a contributing factor [23, 24]. In addition, Yamada pointed out that repeated or persistent stimulation of peripheral T cells would result in the co-expression of both Fas and FasL on T cells, ultimately leading to apoptosis induced by antigen receptor activation [25]. However, whether activation-induced cell death (AICD) or apoptosis of T cells could be reversed [26] or not in the presence of common gamma chain cytokines remains controversial. Our results demonstrated that these Ts cells without cytokines in culture system were prone to apoptosis compared with CD8^+^CD28^+^ T cells, and the combination of cytokines reduced cell death during the expansion process for CD8^+^CD28^−^ T cells (Fig. 3c). Overall, the common gamma chain cytokines appeared to be the critical survival factors in the expansion cultures.

CD8^+^CD28^−^ T cells could be further distinguished into different subpopulations according to relevant immune molecules. Low levels of CD27 on CD8^+^ T cells were associated with CD8^+^ T cell senescence, the expression of CD27 on CD8^+^ T cells has been shown to promote long-term survival of functional effector–memory CD8^+^ T cells [14]. In this research, CD27 was up-regulated on the expanded CD8^+^CD28^−^ Ts cells (Fig. 4), compared with their counterparts in fresh PBMCs, which reflected that the expanded CD8^+^CD28^−^ Ts cells are closer to long-term survival and functional effector–memory T cells. Moreover, these Ts cells developed into central memory T cells—CD44^+^CD62L^+^CCR7^+^—that resided in and traveled between secondary lymphoid tissues. When they reencountered their cognate antigen in secondary lymphoid tissue, they were rapidly activated and mounted an appropriately focused response to their cognate antigens.

Down-regulation of perforin [13] and granzyme B on the in vitro*-*expanded CD8^+^CD28^−^ Ts cells, and low level of FasL expression on the expanded CD8^+^CD28^−^ T cells, shown in Fig. 4, ruled out the possibility of cytotoxicity for these cells as a mechanism of suppression of CD4^+^ T cells proliferation [27, 28], consistent with previous studies of direct cytotoxicity assays [13].

Just as with CD4^+^ Treg cells, several possible mechanisms have been proposed for the suppressive activity of the CD8^+^CD28^−^ Ts cells. It was generally believed that CD8^+^CD28^−^ Ts cells expressed GITR [29, 30], and common γ-chain cytokines were found to significantly upregulate the expression of PD-1 and PD-L1 [31]. Our expanded CD8^+^CD28^−^ Ts cells had no GITR and expressed only low levels of PD-1 and PD-L1 (Fig. 4). Tim-3, however, a relatively new member of negative immunomodulatory molecules closely related to human autoimmune diseases [32], was induced by the common γ chain cytokines in an antigen independent manner [33]. In our investigation, however, blocking Tim-3 with anti-Tim-3 antibody had no effect on the CD4^+^ T cells proliferation, suggesting that Tim-3 did not contribute to the inhibitory effect of these expanded CD8^+^CD28^−^ Ts cells.

## Conclusion

CD8^+^CD28^−^ Ts cells, which were generated by the combination of the common gamma chain cytokines with allogeneic APCs, remained potently suppressive capacity in an allospecific manner in in vivo circumstances. The in vitro expansion of these cells was dependent on several different pathways, including enhancing the proliferation of freshly isolated CD8^+^CD28^−^ T cells, inducing the transformation of CD28^+^ cells to CD28^−^ cells, decreasing the necrosis of CD8^+^CD28^−^ T cells. Phenotypic markers of in vitro expanded CD8^+^CD28^−^ Ts cells might give clues for further investigation of their suppressive mechanisms in future. Anyway, It is of great importance to prove their suppressive effect in a more realistic setting such as a transplantation or autoimmune model which would rather resemble the future situation of clinical application.

## Methods

### Sample collection and PBMCs isolation

Ethical approval for the human studies within this work was obtained from the Ethical Review Board of Southern Medical University. Peripheral blood samples were acquired in heparinized tubes from healthy volunteers with written informed consent in accordance with the Declaration of Helsinki. HLA typing concerning the allelic forms of HLA-A, −B, −DR was determined through a contract of service with molecular assays (Shenzhen YILIFANG Biotech CO.LTO., Shenzhen, China). The peripheral blood mononuclear cells (PBMCs) were isolated from buffy coats of peripheral blood samples by density gradient centrifugation using Ficoll-Hypaque Solution (Tianjin HAOYANG Biological Manufacture CO., LTD., Tianjin, China). Six different groups of donors were screened from 130 volunteers, each group contains 3 different individuals who acted as donor A, −B or -I in subsequent experiments. Donors A, −B and I are fully mismatched in HLA-A, −B, −DR antigen from each other. APCs from donor B were used as priming cells when CD8^+^CD28^−^ Ts cells were expanded in vitro. The representative HLA typing from three different individuals in one group acting as donor A, −B and -I were showed in Table 1 (Numbers represent serological antigens of HLA locus). 
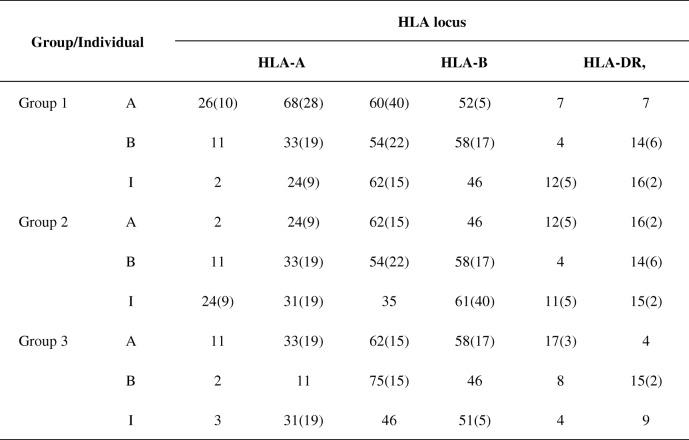

**Table 1:** HLA typing of donor groups with fully mismatched HLA locus used in experiments

Individual		HLA locus					
		HLA-A		HLA-B		HLA-DR,	
Group 1	A	26 (10)	68 (28)	60 (40)	52 (5)	07	07
	B	11	33 (19)	54 (22)	58 (17)	04	14 (6)
	I	2	24 (9)	62 (15)	46	12 (5)	16 (2)
Group 2	A	02	24 (9)	62 (15)	46	12 (5)	16 (2)
	B	11	33 (19)	54 (22)	58 (17)	04	14 (6)
	I	24 (9)	31 (19)	35	61 (40)	11 (5)	15 (2)
Group 3	A	11	33 (19)	62 (15)	58 (17)	17 (3)	4
	B	02	11	75 (15)	46	08	15 (2)
	I	03	31 (19)	46	51 (5)	04	09

The HLA typing of different individuals (Numbers represent serological antigens of HLA locus) are showed. Each group include individual A (for A-CD8+ T cells and A-CD4+ T cells), individual B (for B-APCs) and individual I (for I-APCs), The HLA-A, -B and -DR antigens of individual A, individual B and individual I in the same group are completely mismatched between each other. (Each group of donors whose HLA-A, -B and -DR fully mismatched with each other were selected from over 130 volunteers for independent experiments

### Mice

NOG mice, which were deficient in T and B cells, were purchased from Beijing Vital River Laboratory Animal Technology Co Charles River Laboratories (Chinese agent of Charles River, Beijing, China). All animals were 6- to 8-week old, female, and maintained in the Experimental Animal Center of the Southern Medical University under specific pathogen-free (SPF) conditions. All animal experiments were carried out in accordance with the Guidelines for the Care and Use of Animals established by the Animal Care and Use Committee of Southern Medical University, and approval from the Ethics Committee of Southern Medical University.

### In vitro expansion of CD8^+^CD28^−^ T cells

Cultures for the in vitro generation of CD8^+^CD28^−^ T cells (From individual A) were set up as described previously [13]. Briefly, CD8^+^ T cells were purified from PBMCs of individual A (A-CD8^+^ T cells) by positive selection using the MACS system (Miltenyi Biotec, Bergisch Gladbach, Germany) according to the manufacturer’s instructions. APCs were isolated from HLA-A, −B, −DR mismatched individual B (B-APCs) by depletion of CD2^+^ cells using CD2 microbeads (Miltenyi Biotec, Bergisch Gladbach, Germany). Flow cytometric analysis showed that the purity of sorted cell suspensions was ≥97%. Enriched CD8^+^ T cells (2 × 10^6^ cells) were stimulated with B-APCs (1 × 10^6^ cells) for 9 days in 24-well plates in 2 ml complete medium (RPMI 1640 supplemented with 15% fetal bovine serum) (Gibco, Thermo Fisher Scientific, Waltham, MA, USA) in the presence of IL-2 (PeproTech, Rocky Hill, NJ, USA) at 20 U/ml, IL-7 (PeproTech, Rocky Hill, NJ, USA) at 50 ng/ml, IL-15 (PeproTech, Rocky Hill, NJ, USA) at 50 ng/ml in an incubator at 37 °C and a humidified, 5.5% CO_2_ atmosphere. After 9 days’ incubation, CD28^−^ cells were further enriched by a negative selection strategy, using anti-CD28-PE antibody and anti-PE microbeads combination to remove CD28^+^ cells (Miltenyi Biotec, Bergisch Gladbach, Germany). These CD8^+^CD28^−^ T cells were used for evaluating their suppressive activity.

### In vitro suppression assays

CD4^+^ T cells were isolated from PBMCs of individual A (A-CD4^+^ T cells) by positive selection using the MACS system (Miltenyi Biotec, Bergisch Gladbach, Germany). Freshly purified CD4^+^ T cells were stained with final concentration of 0.5 μM 5-(and 6)-carboxy fluorescein diacetate succinimidyl ester (CFSE; Invitrogen, Thermo Fisher Scientific, Waltham, MA, USA) following the standard procedure. The CFSE-labeled CD4^+^ T cells (5 × 10^4^/well) and in vitro-expanded CD8^+^CD28^−^ T cells (2.5 × 10^4^/well) were seeded into 96-well round bottom plates at a ratio of 1:0.5 within a total volume of 200 μl of complete medium. As stimulators, APCs (5 × 10^4^/well) from individual B (B-APCs) or from HLA-A, −B, −DR fully mismatched indifferent individual I (I-APCs) were also added, respectively. Responder CD4^+^ T cells stimulated with B-APCs cells or I-APCs cells only served as positive controls (B-APC was used as priming cell in vitro to expand CD8^+^CD28^−^T cells from individual A whereas I-APC had never had immune recognition with CD8^+^CD28^−^T cells from individual A in in vitro expanding period). After 7 or 11 days of coculture, the percentage of proliferating CD4^+^ T cells was detected for CFSE dilution by flow cytometry. The suppressive percentage was calculated as follow: %suppression = [1 – (percentage of CD4^+^ T cells proliferation in the presence of CD8^+^CD28^−^ T cells) / (percentage of CD4^+^ T cells proliferation in the absence of CD8^+^CD28^−^ T cells)] *100.

### In vivo suppression assays

Isolation of responder CD4^+^ T cells and APCs was performed in a similar manner as above. 4 × 10^6^ human CD4^+^ T cells were first mixed with an equal number of APCs either from B-APCs or I-APCs, and then combined with 2 × 10^6^ in vitro expanded human CD8^+^CD28^−^ T cells in a total volume of 1.5 ml of PBS. The responder CD4^+^ T cells alone plus B-APCs were used in the same fashion as a positive control. Cell mixture for each group was administered into NOG mice by intraperitoneal injection. Mice were sacrificed on 11th day of post-injection. The spleen was isolated, and half of spleen was homogenized and red cells were lysed with 1× RBC lysis buffer (ebioscience, Thermo Fisher Scientific, Waltham, MA, USA). The absolute number of human CD4^+^ T cells was measured as suppressive activity of CD8^+^CD28^−^ T cells in the presence of donor antigen by flow cytometry. The in vivo experiments in mice were repeated for 3 times, CD8^+^CD28^−^ T cells expanded in vitro were from 3 different groups of volunteers (each group included HLA-A, −B, −DR antigen completely mismatched individuals A, B and I), and were transferred to three batches of NOG mice for each group according to different cellular components.

### Immunohistochemistry

Carrying on in vivo suppression assays, the other half of spleen tissue was fixed immediately in 4% paraformaldehyde, and tissue sections were deparaffinized with xylene and rehydrated through graded ethanol washes. Heat mediated antigen retrieval was conducted by boiling in Tris/EDTA buffer (pH 9.0) for 5 min. The slides were incubated with 3% hydrogen peroxide for 20 min to eliminate endogenous peroxidase and then with 10% normal goat serum at room temperature in order to block any non-specific binding. After removing excess blocking buffer, the following primary antibodies, concerning rabbit anti-human CD8 antibody (ab93278, Abcam, Cambridge, UK) and mouse anti-human CD4 antibody (ZM-0418, ZSGB-BIO, Beijing, China), were diluted to the manufacturer’s recommendations and incubated in a humidified chamber at 4 °C overnight. The HRP-conjugated goat anti-rabbit IgG antibody (PV-6001, ZSGB-BIO, Beijing, China) and goat anti-mouse IgG antibody (PV-6002, ZSGB-BIO, Beijing, China) were used as the secondary antibody at 37 °C for 30 min. Finally, staining of the tissue sections was performed with an enhanced HRP-DAB chromogenic substrate kit (TIANGEN Biotech CO., LTD., Beijing, China). The sections were then counterstained with hematoxylin and visualized under a light microscope (Nikon, Tokyo Japan).

### Flow cytometry analysis

Flow cytometry analysis was performed on those cells harvested from the culture under different conditions by using standard techniques. Briefly, cell suspensions were collected, washed and stained with fluorescently labeled antibodies as indicated. The antibodies used in these studies were as follows: PerCP-Cy5.5-conjugated anti-CD8a (RPA-T8); allophycocyanin-conjugated anti-CD28 (CD28.2), CD4 (SK3); FITC-conjugated anti-CD122 (TU27), CD27 (0323), CCR7 (3D12), PD-1 (MIH4), CD2 (RPA-2.10); PE-conjugated anti-CD215 (eBioJM7A4), CD132 (TUGh4), CD44 (IM7), GITR (eBioAITR), PD-L1 (MIH18), Tim3 (F38-2E2), Fas ligand (NOK-1), Granzyme B (GB11), CD45 (HI30). Isotype-matched, FITC- or PE-conjugated mAbs, specific for non-relevant antigens, were used as negative controls. The antibodies, except CD132 purchased from Biolegend (San Diego, CA, USA), were all purchased from eBioscience (Thermo Fisher Scientific, Waltham, MA, USA). For apoptosis/necrosis staining, the percentage of necrotic cells (Annexin V^+^ 7-AAD^+^) in CD8^+^CD28^+^ and CD8^+^CD28^−^ T cells were assessed by flow cytometry after incubation with FITC-conjugated Annexin V (Nanjing Keygen Biotech CO., LTD., Nanjing, China) and 7-AAD (Thermo Fisher Scientific, Waltham, MA, USA) following the manufacturer’s instructions. Flow cytometry data were acquired by using an LSRFortessa flow cytometer (BD Biosciences, San Jose, CA, USA). Data were analyzed by using the FlowJo vX.0.7 software.

### Method of sacrifice

Mice were sacrificed by inhalation of CO_2_ at scheduled time post intraperitoneal injection. Specifically, the mouse was put into a euthanasia cage, then filling CO_2_ gas, the mouse would soon lose consciousness and die within 3 ~ 5 min. Took out the mouse, the spleen was harvested for next experiment in clean bench.

### Statistical analysis

Statistical analysis was carried out on SPSS version 22 software. For comparison of different groups where appropriated, independent-samples T test and nonparametric test were used for determining statistical significance. Statistical graphs were performed by using the GraphPad Prism version 5.01 software.

## Data Availability

The datasets used and/or analyzed during the current study are available from the corresponding author on reasonable request.

## Abbreviations

APCs: Antigen presenting cells
IL: Interleukin
PBMCs: Peripheral blood mononuclear cells
HLA: Human leukocyte antigen
CFSE: 5-(and 6)-carboxy fluorescein diacetate succinimidyl ester
Tregs: Regulatory T cells
FasL: Fas ligand
PD-1: Programmed death-1
Tim-3: T cell Ig mucin domain containing molecule 3
GITR: Glucocorticoid-induced TNF receptor
IL-2R: IL-2 receptor
